# Financial Distress and Its Determinants in Rheumatoid Arthritis

**DOI:** 10.1002/acr.25670

**Published:** 2025-12-15

**Authors:** Amber Brown Keebler, Yunju Im, Sofia Pedro, Ted R. Mikuls, Edward S. Peters, Kaleb Michaud

**Affiliations:** ^1^ Division of General Internal Medicine, Department of Internal Medicine University of Nebraska Medical Center Omaha; ^2^ Department of Biostatistics College of Public Health, University of Nebraska Medical Center Omaha; ^3^ FORWARD, The National Databank for Rheumatic Diseases Wichita Kansas; ^4^ Division of Rheumatology, Department of Internal Medicine University of Nebraska Medical Center Omaha; ^5^ Medicine and Research Services, VA Nebraska‐Western Iowa Health Care System Omaha Nebraska; ^6^ Department of Epidemiology College of Public Health, University of Nebraska Medical Center Omaha

## Abstract

**Objective:**

To quantify the degree of financial distress and identify its determinants in adults with rheumatoid arthritis (RA) given the frequent prolonged use of expensive disease‐modifying therapies.

**Methods:**

We identified adults enrolled in the FORWARD databank with either RA or noninflammatory musculoskeletal disease (NIMSKD) completing the Functional Assessment of Chronic Illness Therapy–Comprehensive Score for Financial Toxicity (FACIT‐COST) questionnaire. In this cross‐sectional study, FACIT‐COST was analyzed as a continuous (higher score indicates less financial distress) and binary variable (presence of financial distress with threshold <26). Least Absolute Shrinkage and Selection Operator (LASSO) was applied to linear and logistic regression to select covariates for inclusion in multivariable models.

**Results:**

Participants with RA (n = 2,277) had lower FACIT‐COST scores, indicating greater financial distress, than those with NIMSKD (n = 1,340) (mean of 30.2 ± SD 9.4 vs mean of 34.0 ± SD 8.4; unadjusted *P* < 0.001). Assessed as a binary outcome, financial distress was more frequent in participants with RA than participants with NIMSKD (29% vs 15%; unadjusted *P* < 0.001). Differences in financial distress by diagnosis persisted following multivariable adjustment. Among those with RA, determinants identified in multivariable models included depression (adjusted odds ratio 1.12; 95% confidence interval 1.09–1.16) and disease severity.

**Conclusion:**

Financial distress is prevalent in adults with RA and appears to be greatest in those with comorbidities, specifically depression, identifying a potential area for intervention. Notably, expensive biologic or targeted synthetic disease‐modifying antirheumatic drugs were not associated with FACIT‐COST scores.

## INTRODUCTION

Health care costs and financial distress are recognized as significant barriers to successfully managing chronic health conditions. Financial distress, often referred to as financial toxicity, is defined as the adverse effect of health care costs on a patient's financial well‐being.^1^ Health outcomes of decreased medication and treatment adherence in chronic disease are associated with financial distress. In a study by Khera et al,^2^ financial distress was described among patients with cardiovascular disease. In a study by Patel et al,^3^ the Comprehensive Score for Financial Toxicity was validated in patients with diabetes. It has been most robustly studied in patients undergoing treatment for cancer. Among young adults with cancer, it is also associated with unhealthy coping such as skipping treatments and lower levels of psychological well‐being.^4^ Overall, the perception of difficulty paying bills is associated with lower health care use.^5^ Moreover, higher levels of financial distress are also associated with an increased frequency of depression and anxiety, which may serve to mediate the relationship between financial distress and poor patient outcomes.^4^, ^6^

**SIGNIFICANCE & INNOVATIONS**

- To our knowledge, this report is among the first to describe the prevalence of financial distress in adults with rheumatic disease in the US.
- Approximately one‐third of participants with rheumatoid arthritis (RA) demonstrate evidence of financial distress with a prevalence that is approximately twice that of those with noninflammatory musculoskeletal disease.
- Disease severity, depression, and other comorbidities are associated with financial distress in RA.
- Receipt of expensive advanced therapies in RA are not strongly associated with the presence or severity of financial distress.

Although recognized as important in facilitating shared decision making, financial hardship, socioeconomic status, and financial distress are not routinely evaluated or discussed in day‐to‐day clinical practice. Though health insurance status is routinely evaluated, insurance coverage alone does not protect against financial distress.^6^ In a study by Perez et al,^7^ only 50% of US internists who responded to a survey reported having frequent cost conversations with their patients, though 75% reported using cost of care as a factor in guiding clinical decision‐making. Strikingly, patients report high levels of interest in having these conversations with health care providers. In a study by Henrikson et al,^8^ most respondents preferred their provider to have a cost conversation with them, and 75% felt comfortable with having these discussions.

Despite the importance of cost of care and its communication to both patients and health care providers, comprehensive examinations of financial distress in rheumatic disease, including rheumatoid arthritis (RA), have been limited to date. This knowledge gap is highly relevant as RA accounts in the US for 257,884 disability‐adjusted life‐years, and RA‐related health care costs have risen markedly in recent years.^9^ For a patient with RA receiving advanced therapies, for example, total annual per‐person direct medical costs have been estimated to exceed $36,000.^10^

Given these prevailing knowledge gaps, we sought to examine the impact of financial distress in adults with rheumatic disease, including individuals with RA, and in those with noninflammatory musculoskeletal disease (NIMSKD). Using data from a well‐characterized population of rheumatic disease participants, we sought to test the hypotheses that financial distress is more prevalent and more severe in RA than NIMSKD. Moreover, we sought to identify factors predictive of financial distress beyond rheumatic disease status. We tested the additional hypotheses that among those with RA, the use of advanced biologic or targeted synthetic therapies along with comorbid conditions, particularly the presence of depression and obesity, would be associated with higher levels of financial distress independent of other factors.

## PATIENTS AND METHODS

### Study design and participants

This cross‐sectional study was performed using data from the FORWARD databank, previously known as The National Databank for Rheumatic Diseases.^11^ FORWARD is an ongoing longitudinal observational registry that collects patient‐reported outcomes on multiple rheumatic conditions (eg, RA, NIMSKD, and other inflammatory/autoimmune conditions) using biannual questionnaires covering a wide range of information such as sociodemographic factors, rheumatologic diagnoses, comorbidities, treatments, symptoms, health care utilization, and other long‐term outcomes as previously detailed.^12^, ^13^, ^14^ Participating US rheumatology clinics aid in the recruitment of registry participants and confirmation of primary rheumatologic diagnoses.^11^

### Inclusion and exclusion criteria

This study included databank participants with either RA or NIMSKD and was limited to individuals with data available for the primary outcome as detailed below. Diagnoses considered as NIMSKD included predominantly chronic musculoskeletal diseases including Dupuytren's contracture (68%) and osteoarthritis (32%) but excluding fibromyalgia.^15^, ^16^ Participants with other forms of systemic inflammatory disease (eg, systemic lupus erythematosus, spondyloarthritis, crystal arthropathy) were excluded.

### Outcomes

Financial distress experienced by participants was measured using the validated 12‐item Comprehensive Score for Financial Toxicity–Functional Assessment of Chronic Illness Therapy (FACIT‐COST) instrument and served as the primary outcome for this analysis.^1^ This tool includes measurement of effect on dominant themes of finance, resources, affect, and coping, as well as effects on family relationships. The score has established correlations with perceived support from family and friends, age, savings, and household income.^17^, ^18^ Although originally utilized in oncology, the FACIT‐COST measure has been validated in other chronic disease populations, demonstrating both internal consistency and predictive validity.^19^ Response options for each item are provided on a 5‐point Likert scale with each response scored from 0 to 4 based on responses of “not at all,” “a little bit,” “somewhat,” “quite a bit,” and “very much,” respectively. The sum of individual item scores is then used as a severity score (range of 0 to 48), with lower scores indicating higher financial distress. For study purposes, the FACIT‐COST score was computed if at least 6 of the 12 questions were answered per developer scoring guidance.^20^ In addition to examining the severity score as a quantitative measure, we also examined a binary categorization defining the presence of financial stress by scores <26 and absence of financial distress by scores of ≥26 as utilized in other studies.^20^, ^21^, ^22^

### Covariables

In addition to rheumatologic disease status (RA vs NIMSKD), other patient‐reported factors were examined as possible determinants of treatment‐related financial distress. Depression was assessed using the eight‐item Patient Health Questionnaire (PHQ‐8) depression scale, a standardized, well‐validated diagnostic and severity measure for depressive disorders.^23^ This scale includes the criteria for depression as defined by the Diagnostic and Statistical Manual of Mental Disorders V, excluding suicidal ideation, as related questions are not included on FORWARD questionnaires. PHQ‐8 scores range from 0 to 24 with scores ≥10 indicative of moderate‐to‐severe depression.^23^

Obesity was assessed using body mass index (BMI: kg/m^2^) calculated from self‐reported height and weight values at the time of completing the FACIT‐COST questionnaire. BMI categories included underweight (<20 kg/m^2^), normal (20–24.9 kg/m^2^, referent category), overweight (25–29.5 kg/m^2^), class 1 obesity (30–34.9 kg/m^2^), class 2 obesity (35–39.5 kg/m^2^), and class 3 obesity (≥40 kg/m^2^).

Additional variables examined as possible determinants of financial distress included the following self‐reported sociodemographic factors: sex, race (White vs non‐White), age, college education or higher (yes/no), total annual household income, marital status (married vs not married), insurance coverage (Medicaid or Medicare: yes/no), and employment status (employed/retired vs not employed/retired). Clinical variables included Rheumatic Disease Comorbidity Index (RDCI),^24^ physical disability quantified using the Health Assessment Questionnaire Disability Index (HAQ‐DI),^25^ Patient Global Assessment (PGA; 0–10),^26^, ^27^ and patient‐reported disease activity (Patient Activity Scale [PAS], range from 0 to 10).^26^ Individual comorbidities composing the RDCI (cancer, stroke, cardiovascular disease, diabetes, fracture, gastrointestinal conditions, neurologic disease, respiratory disease, renal impairment, liver disease, and depression), glucocorticoid use, and disease‐modifying antirheumatic drug (DMARD) treatment were also included in analyses. Where relevant, DMARDs were categorized as conventional synthetic DMARD (csDMARD), tumor necrosis factor inhibitor (TNFi), non‐TNFi biologic, or JAK inhibitor (JAKi). Participants with a NIMSKD diagnosis were excluded if they were on a DMARD or prednisone to reduce the risk of selection bias.

### Statistical analysis

Descriptive statistics were used to describe characteristics of the overall sample and make comparisons between the RA and NIMSKD groups. Diagnosis groups were further subdivided by presence of financial distress (threshold of FACIT‐COST score of <26); within group comparisons were performed using *t*‐tests and chi‐square tests as appropriate.

Univariate linear and logistic regression analyses were initially undertaken to examine marginal associations between financial distress (both continuous and binary outcomes, respectively) and each covariate. Least Absolute Shrinkage and Selection Operator (LASSO) was then used to select statistically significant variables associated with financial distress. LASSO is a linear regression method that estimates the regression coefficients by maximizing the log‐likelihood function with a constraint imposed on the sum of the absolute values of regression coefficients.^28^ To adequately account for confounding effects and given our focus on disease status, age, sex, total annual household income, RDCI, and RA status were fixed and not subject to LASSO variable selection. All other covariates were available for LASSO selection. As LASSO does not provide the SEs required for computing confidence intervals (CIs), we subsequently used a double‐LASSO approach in the overall model to provide 95% CIs.^28^, ^29^ For RA specific models, estimates and 95% CIs were obtained by following initial LASSO variable selection with standard regression.

For missing values, where available, a most recent value carried forward or backward approach was used. Missing sex status was also imputed using an available registry variable for “menstrual cycles.” Missing HAQ‐DI and PAS values were replaced using HAQ‐II or PAS‐II scores, respectively, when available in the same phase questionnaire. Beyond these, a complete‐case approach was used, excluding individuals with missing covariate data from relevant analyses. Less than 10% of participants were excluded from multivariable models due to missing covariates.

All data analyses were conducted using Stata version 17^30^ (StataCorp) and R (R Foundation) glmnet.^31^ Values of *P* < 0.05 were considered statistically significant.

### Ethical approval

This study was considered exempt by the University of Nebraska Institutional Review Board (IRB; number 0093‐07‐EX) based on previous approval from the Ascension Via Christi Hospitals Wichita, Inc, IRB (IRB number: IRB00001674). Individuals participating in FORWARD undergo informed consent before data collection.

## RESULTS

### Participant characteristics

A total of 3,617 participants were included in the sample and analyzed in two diagnosis groups, RA (n = 2,277) and NIMSKD (n = 1,340). Characteristics of those with a diagnosis of RA or NIMSKD are shown in Table 1. Both RA and NISMKD similarly comprised participants with older ages and predominantly of White race, reflective of the overall FORWARD participant population. Though both groups were predominantly female, the proportion was higher in RA (84%) compared to NIMSKD (67%). Retirement status as well as Medicaid and Medicare insurance status were also similar between the two diagnosis groups. Compared to NIMSKD, RA participants had a lower total household income, were less likely to have a college education, and were less likely to be employed. Among participants with RA, 49.7% were on biologic treatment modalities and 59.5% were on csDMARDs.

**Table 1:** Characteristics of study participants*

Variables	RA (n = 2,277)	NIMSKD (n = 1,340)
Demographics		
Age, y	69.8 (10.7)	68.8 (10.2)
Total household income, US $1,000	73.4 (42.1)	90.8 (41.5)
College education, %	54.1	73.2
Employed, %	20.1	28.2
Married, %	64.1	71.3
Retired, %	60.1	62.0
Female, %	83.9	66.6
White, %	90.8	96.8
Medicaid, %	4.3	2.6
Medicare, %	70.1	66.0
Quality of life and disease‐related severity		
HAQ‐II (0–3)	0.9 (0.7)	0.4 (0.6)
Patient Global (0–10)	3.6 (2.5)	2.1 (2.4)
PAS (0–10)	3.5 (2.2)	1.9 (2.1)
Comorbidities		
RDCI (0–9)	2.0 (1.7)	1.7 (1.6)
BMI, kg/m^2^	28.4 (7.0)	26.6 (6.6)
Cancer, %	6.0	6.4
Stroke, %	4.5	5.8
Cardiovascular, %	14.5	12.4
PHQ‐8 (0–24)	4.4 (4.4)	3.1 (3.7)
Depression, %	14.2	10.2
Diabetes, %	10.3	8.4
Liver disease, %	3.3	3.4
Treatments		
csDMARD, %	59.5	–
Biologic, %	49.7	1.1
TNFi, %	28.5	0.5
Non‐TNFi, %	22.1	0.6
JAKi, %	9.4	0.1

* Values are mean (SD) unless otherwise indicated. Mean standard difference is >0.1 for all variables except for the following: age, retired, Medicaid, Medicare, cancer, stroke, cardiovascular, diabetes, and liver disease. Stroke includes transient ischemic attack and other prior cerebrovascular accidents; cardiovascular includes heart failure and myocardial infarction, but not high blood pressure. BMI, body mass index; csDMARD, conventional synthetic disease‐modifying antirheumatic drugs; HAQ‐II, Health Assessment Questionnaire II; JAKi, JAK inhibitor; NIMSKD, noninflammatory musculoskeletal disease; PAS, Patient Activity Scale; PHQ‐8, Patient Health Questionnaire 8; RA, rheumatoid arthritis; RDCI, Rheumatic Disease Comorbidity Index; TNFi, tumor necrosis factor inhibitor.

### FACIT‐COST scores in RA vs NIMSKD

**Figure 1:**
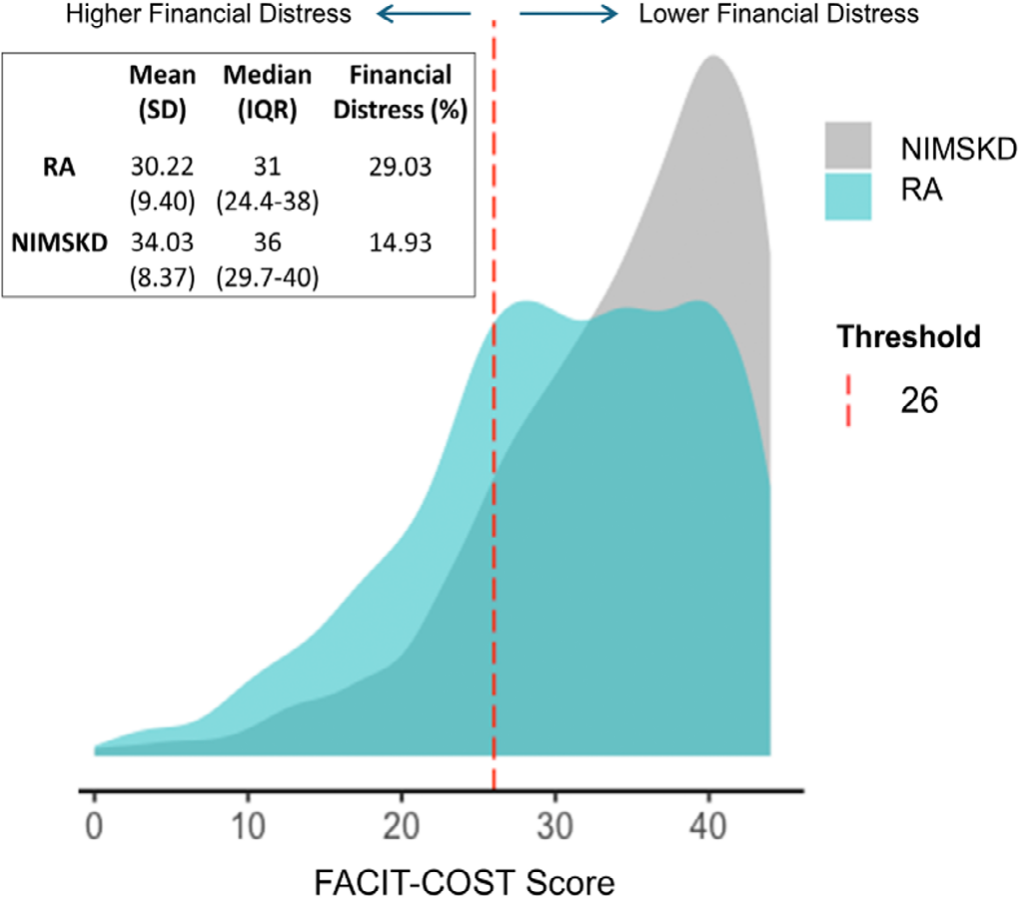
Distribution of FACIT‐COST scores for people with RA and NIMSKD. Lower scores as seen in RA are indicative of higher financial distress. FACIT‐COST, Comprehensive Score for Financial Toxicity–Functional Assessment of Chronic Disease; IQR, interquartile range; NIMSKD, noninflammatory musculoskeletal disease; RA, rheumatoid arthritis.

**Figure 2:**
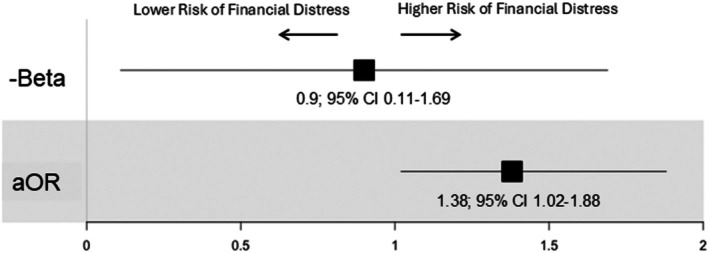
Diagnosis of RA associated with risk of financial distress in both linear and logistic models. Linear model represented as −beta. Logistic model represented as OR; both models indicate that a diagnosis of RA is associated with higher risk of financial distress. aOR, adjusted OR; CI, confidence interval; OR, odds ratio; RA, rheumatoid arthritis.

In unadjusted analyses, RA participants had lower FACIT‐COST scores (mean ± SD: 30.2 ± 9.4) compared to participants with NIMSKD (34.0 ± 8.4) indicative of greater financial distress in RA (*P* < 0.001 for comparison; Figure 1). The presence of high financial distress (defined as a FACIT‐COST score <26) was also more frequent in RA than NIMSKD (29% vs 15%; unadjusted *P* < 0.001) (Figure 1). After application of LASSO and accounting for covariates, associations of RA diagnosis (vs NIMSKD) was significant in the multivariable linear regression model (−β 0.9; 95% CI 0.11–1.69) though not in the logistic model (adjusted odds ratio [aOR] 1.38; 95% CI 1.02–1.88) (Figure 2). In these models, covariates associated with increased prevalence and/or severity of financial distress included RDCI, PAS, and PHQ‐8, whereas higher household income and older age were independently associated with lower financial distress (Supplemental Table 1). Higher BMI and being retired were significantly associated with higher severity but not prevalence.

### Determinants of financial distress in RA

Subsequent univariable and multivariable analyses were conducted to identify determinants of financial distress only in individuals with RA. In univariable analysis, Table 2 presents the characteristics that were associated with the presence of financial distress (Supplemental Table 2 similarly shows parallel analyses for NIMSKD). Consistent with our a priori hypothesis, those with financial distress had a higher prevalence of multiple comorbid conditions (including depression) and were slightly more likely to be receiving JAKi advanced therapy and less likely to be receiving csDMARDs. Similar results were observed examining univariable associations of clinical factors with FACIT‐COST score.

**Table 2:** RA with and without FD*

Variables	FD present: score <26 (n = 661)	FD NOT present: score ≥26 (n = 1,616)	*P* value
Demographics			
Age, y	67.2 (10.6)	70.8 (10.6)	**
Income, US $1,000	57.7 (39.5)	79.9 (41.4)	**
College education, %	44.7	57.9	**
Employed, %	22.6	19.1	0.08
Married, %	58.7	66.3	*
Retired, %	45.1	66.3	**
Female, %	89.9	81.5	**
Caucasian, %	87.5	92.2	*
Medicaid, %	9.6	2.1	**
Medicare, %	61.6	73.5	**
Quality of life and disease‐related severity			
HAQ‐II (0–3)	1.25 (0.71)	0.81 (0.71)	**
Patient Global (0–10)	4.7 (2.3)	3.1 (2.4)	**
PAS (0–10)	4.6 (2.1)	2.0 (2.1)	**
Comorbidities			
RDCI (0–9)	2.6 (1.8)	1.8 (1.6)	**
BMI, kg/m^2^	29.0 (7.9)	27.7 (6.4)	**
Cancer, %	7.6	5.3	<0.05
Stroke, %	6.6	3.7	*
Cardiovascular, %	17.4	13.4	<0.05
PHQ‐8 (0–24)	7.2 (5.3)	3.3 (3.4)	**
Depression, %	26.7	9.1	**
Diabetes, %	16.8	7.6	**
Liver disease, %	4.7	2.7	<0.05
Treatment			
csDMARD, %	55.5	61.1	<0.05
Biologic, %	48.1	50.4	0.35
TNFi, %	26.8	29.1	0.28
Non‐TNFi, %	22.2	22.0	0.96
JAKi, %	12.1	8.2	*

* The values are mean (SD) unless otherwise indicated. Stroke includes transient ischemic attack and other prior cerebrovascular accidents; cardiovascular includes heart failure and myocardial infarction, but not high blood pressure. BMI, body mass index; csDMARD, conventional synthetic disease‐modifying antirheumatic drugs; FD, financial distress; HAQ‐II, Health Assessment Questionnaire II; JAKi, JAK inhibitor; PAS, Patient Activity Scale; PHQ‐8, Patient Health Questionnaire 8; RA, rheumatoid arthritis; RDCI, Rheumatic Disease Comorbidity Index; TNFi, tumor necrosis factor inhibitor.

* *P* < 0.01.

** *P* < 0.001.

**Figure 3:**
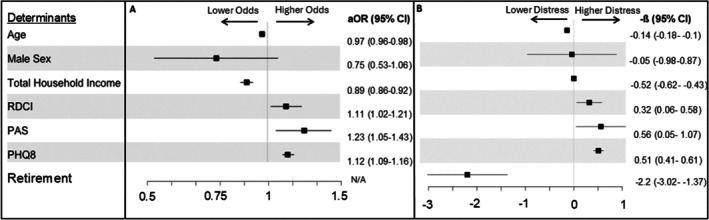
Determinants of financial distress in people with RA. (A) Logistic model with aOR found RDCI, PAS, and PHQ8 scores were associated with higher odds of financial distress. (B) Linear model with beta coefficients found that RDCI, PAS, and PHQ8 scores were associated with a higher financial distress. Retirement had a significant association with lower financial distress. aOR, adjusted odds ratio; CI, confidence interval; N/A, not applicable; PAS, Patient Activity Scale; PHQ8, Patient Health Questionnaire 8; RDCI, Rheumatic Disease Comorbidity Index; RA, rheumatoid arthritis.

After applying LASSO, independent determinants of financial distress in RA included younger age (aOR 0.97; 95% CI 0.96–0.98), lower total household income (aOR 0.89; 95% CI 0.86–0.92), higher RDCI score (aOR 1.11; 95% CI 1.02–1.21), higher PAS score (aOR 1.23; 95% CI 1.05–1.43), and higher PHQ‐8 score (aOR 1.12; 95% CI 1.09–1.16). Similar determinants were again identified in separate multivariable analyses examining FACIT‐COST score with LASSO selection of the additional variable of being retired (−β −2.2; 95% CI −3.02 to 1.37; Figure 3).

## DISCUSSION

In this large observational study, we describe the prevalence and determinants of US‐wide financial distress in adults with RA. As anticipated, a significant proportion of participants have financial distress, emphasizing the need for identifying associated modifiable risk factors, though given the adjusted regression models, it was unlikely due to the higher use of expensive treatment modalities as originally hypothesized. This is notable as previous studies have clearly shown medication expenses compose a significant proportion of steep medical costs in the treatment of RA.^10^ Interestingly, this suggests that factors other than potential treatment costs are contributing to financial distress.

Use of a FACIT‐COST threshold score of 26 has been reported in oncology literature in patients with breast, lung, and colorectal cancer.^21^, ^22^ Prevalence of financial distress using this method demonstrated proportions of 45%^21^ and 77%.^22^ In comparison, approximately 29% of participants with RA in our study met a comparable threshold for financial distress; this lower prevalence can be explained by the significant disparity in medical costs in cancer treatment versus RA treatment. RA specific direct costs compose a substantial proportion of medical costs ranging between $3,723 and $20,262 per annum.^10^ However, cancer far exceeds RA in out‐of‐pocket costs, ranging $2,160 to $31,200^32^ versus $4,801^33^ per annum, respectively.

We observed among the FORWARD participants determinants of financial distress including decreased quality of life, comorbidities including obesity, and depression. Similar to other studies, multiple comorbidities and decreased quality of life have been consistently associated with increased costs and predictive of more financial distress.^34^ Previous studies have also demonstrated a pervasive association of financial distress with mental health diagnoses including depression and anxiety.^4^, ^6^ Strikingly, although unsurprising, depression is the most consistent association with financial distress in our analysis, regardless of diagnosis; the relationship between the two should strongly be considered for future longitudinal studies.

Measures indicative of financial status are associated with financial distress as demonstrated in the use of the FACIT‐COST measure in both cancer and chronic disease.^3^, ^19^, ^21^, ^22^ Our findings are consistent with these previous studies, as we identified a significant inverse association between total household income and financial distress. Unexpectedly, retirement status had a significant negative association with financial distress. In contrast, employment status had a significant positive association with financial distress when FACIT‐COST score was analyzed as a continuous variable. This is in contrast to a study by Weismann et al,^35^ who reported that employment was associated with lower financial distress; in addition, older age was associated with higher financial distress. These differences are likely due to the participants’ widely diverse medical diagnoses and that their study purposefully excluded those aged 65 years or greater, leaving a gap in knowledge of the experience of financial distress for those more likely to be retired. In our study, younger age was more likely to be associated with financial distress, though participants were on average older than 65 years of age. Weismann et al also found, similarly to our results, that lower income and chronic health conditions were associated with financial distress. In our study, secondary analyses demonstrated retired patients had a significantly higher income than those who were not retired. Thus, retirement is a surrogate marker for financial security and wealth, subsequently demonstrating a negative association with financial distress.

Given the prevalence of financial distress in RA, there are significant implications for studying clinical practice implementation methods. Sloan et al^36^ recommended routinely including out‐of‐pocket costs in patient encounters, going so far as to screen for financial hardship even if not suspected. Assessment of financial hardship would appropriately broaden our clinical approach to social determinants of health in routine clinical care. Some studies have further shown that the use of a financial navigator significantly decreased health care costs and financial distress.^37^, ^38^

Though our study provides insight into financial distress in people with rheumatic disease and its associated determinants, there are some limitations. There is less generalizability to non‐White, less educated, and younger affected populations, an issue somewhat common to research registries that have participation/selection bias. As a patient‐reported database, FORWARD is subject to missing data. However, given the low overall rate of missingness and the use of complete‐case analysis for all inferential results, we believe the findings are robust and not meaningfully affected by missing data. As this is a cross‐sectional observational study, we are not able to establish causal inference and some unmeasured confounding may be present. The relationship across depression, financial distress, and disease severity is complex with multiple mediating factors. Future studies should be designed to prospectively determine causal direction and include a more diverse population with respect to race and ethnicity as well as socioeconomic status. Finally, it is unclear if the threshold score of the FACIT‐COST measure is most appropriate, as it was determined in other nonrheumatic cohorts. However, a threshold score could be more meaningful and clinically useful due to ease of interpretation and ability to take immediate actionable steps.^21^, ^22^

In conclusion, we observed that financial distress is prevalent in individuals with RA. Comorbidities including depression and measures of disease severity are significantly associated with financial distress. To our knowledge, this is one of the first studies describing financial distress in a large sample of adults with RA in the US. It highlights the relationship of financial distress with overall health and mental well‐being. These findings should motivate future prospective studies to elucidate causality and evaluate the utility for clinical implementation.

## AUTHOR CONTRIBUTIONS

All authors contributed to at least one of the following manuscript preparation roles: conceptualization AND/OR methodology, software, investigation, formal analysis, data curation, visualization, and validation AND drafting or reviewing/editing the final draft. As corresponding author, Dr Michaud confirms that all authors have provided the final approval of the version to be published and takes responsibility for the affirmations regarding article submission (eg, not under consideration by another journal), the integrity of the data presented, and the statements regarding compliance with institutional review board/Declaration of Helsinki requirements.

## Supporting information


**Disclosure Form**:

**Supplemental Table 1** Overall Multivariable Regression Models (RA and NIMSKD combined)
**Supplemental Table 2a**: Characteristics of Participants with NIMSKD by Financial Distress Status
**Supplemental Table 2b**: NIMSKD Multivariable Regression Models
