# Supplementary material for: Financial Distress and Its Determinants in Rheumatoid Arthritis
Source: Arthritis Care Res (Hoboken). 2025 Dec 15;78(5):601–8. doi: 10.1002/acr.25670 (PMC13116011; doi:10.1002/acr.25670)
Supplement: Supplementary file 2 — Supplemental Table 1 Overall Multivariable Regression Models (RA and NIMSKD combined) Supplemental Table 2a: Characteristics of Participants with NIMSKD by Financial Distress Status Supplemental Table 2b: NIMSKD Multivariable Regression Models [file ACR-78-601-s001.docx]

**Supplemental Table 1: Overall Multivariable Regression Models (RA and NIMSKD combined)**

| **Variables** | **-Beta (95% CI)** | **OR (95% CI)** |
| --- | --- | --- |
| RA Diagnosis | 0.9 (0.11, 1.69) | 1.38 (1.102, 1.88) |
| Age | -0.13 (-0.16, -0.09) | 0.96 (0.95, 0.97) |
| Male Sex | -0.05 (-0.69, 0.59) | 0.81 (0.61, 1.06) |
| Income (US$1000) | -0.49 (-0.56, -0.42) | 0.89 (0.86, 0.91) |
| RDCI | 0.32 (0.12, 0.53) | 1.11 (1.03, 1.20) |
| PAS | 0.49 (0.01, 0.88) | 1.21 (1.06, 1.38) |
| PHQ8 | 0.52 (0.44, 0.59) | 1.13 (1.10, 1.16) |
| Retired | -2.25 (-2.88, -1.63) | ------ |

Grey shading indicates fixed variables in the LASSO model.

Abbreviations: BMI, body mass index; NIMSKD, non-inflammatory musculoskeletal disease; PAS, Patient Activity Scale; Patient Global, Patient Global Assessment; PHQ8, Patient Health Questionnaire 8; RA, rheumatoid arthritis; RDCI, Rheumatic Disease Comorbidity Index

**Supplemental Table 2a: Characteristics of Participants with NIMSKD by Financial Distress Status**

| **Variables** | **Financial Distress Present (<26)** | **Financial Distress NOT Present (≥26)** | **p-value** |
| --- | --- | --- | --- |
|  | **n=200** | **n=1140** |  |
| *Demographics* | | | |
| Age (years) | 65.06 (10.47) | 69.45 (10.04) | < 0.001 |
| Total Household Income (US$1000) | 68.88 (40.93) | 94.77 (40.32) | < 0.001 |
| College Education, % | 56.30 | 76.10 | < 0.001 |
| Employed, % | 33.10 | 27.40 | 0.13 |
| Married, % | 55.40 | 74.10 | < 0.001 |
| Retired, % | 43.10 | 65.10 | < 0.001 |
| Female, % | 78.70 | 64.60 | < 0.001 |
| Caucasian, % | 93.20 | 97.40 | < 0.01 |
| Medicaid, % | 5.30 | 2.20 | 0.07 |
| Medicare, % | 56.50 | 67.60 | < 0.05 |
| *Quality of Life and Disease Related Severity* | | | |
| HAQ-II (0-3) | 0.78 (0.71) | 0.37 (0.52) | < 0.001 |
| Patient Global (0-10) | 3.84 (2.67) | 1.82 (2.18) | < 0.001 |
| PAS (0-10) | 3.48 (2.37) | 1.67 (1.86) | < 0.001 |
| *Comorbidities* | | | |
| RDCI (0-9) | 2.42 (1.81) | 1.57 (1.50) | < 0.001 |
| BMI (kg/m^2^) | 28.96 (7.79) | 26.14 (6.21) | < 0.001 |
| Cancer, % | 11.00 | 5.50 | < 0.01 |
| Stroke, % | 9.50 | 5.20 | < 0.05 |
| Cardiovascular, % | 21.50 | 10.80 | < 0.001 |
| PHQ-8 (0-24) | 6.51 (5.17) | 2.53 (3.05) | < 0.001 |
| Depression, % | 23.00 | 7.90 | < 0.001 |
| Diabetes, % | 12.50 | 7.70 | < 0.05 |
| Liver Disease, % | 6.50 | 2.90 | < 0.05 |

*Values are mean (SD) unless otherwise indicated.

Abbreviations and clarifications: NIMSKD, non-inflammatory musculoskeletal disease; HAQ-II, Health Assessment Questionnaire-II; PAS, Patient Activity Scale; RDCI, Rheumatic Disease Comorbidity Index; BMI, body mass index; Stroke includes transient ischemic attack and other prior cerebrovascular accidents; Cardiovascular includes heart failure, myocardial infarction, but not high blood pressure; PHQ-8, Patient Health Questionnaire 8;

**Supplemental Table 2b: NIMSKD Multivariable Regression Models**

| **Variables** | **- Beta (95% CI)** | **OR (95% CI)** |
| --- | --- | --- |
| Age | -0.12 (-0.16, -0.07) | 0.95 (0.94, 0.96) |
| Male Sex | 0.05 (-0.80, 0.90) | 0.71 (0.51, 1.00) |
| Income (US$1000) | -0.46 (-0.57, -0.36) | 0.88 (0.86, 0.91) |
| RDCI | 0.39 (-0.11, 0.67) | 1.16 (1.08, 1.28) |
| PAS | 0.53 (0.11, 0.94) | 1.21 (1.13, 1.28) |
| PHQ-8 | 0.53 (0.41, 0.66) | 1.13 (1.09, 1.16) |
| Retired | -2.46 (-3.40, -1.53) | ----- |
| BMI (kg/m^2^) | 0.11 (0.05, 0.18) | ----- |
| Patient Global | 0.31 (-0.05, 0.66) | ----- |

Grey shading indicates fixed variables in the LASSO model.

Abbreviations: BMI, body mass index; NIMSKD, non-inflammatory musculoskeletal disease; PAS, Patient Activity Scale; Patient Global, Patient Global Assessment; PHQ8, Patient Health Questionnaire 8; RA, rheumatoid arthritis; RDCI, Rheumatic Disease Comorbidity Index
